# Case of Abnormal Fœtation

**Published:** 1876-07

**Authors:** John Coston


### CASE OF ABNORMAL FŒTATION.

By JOHN COSTON, M.D.

I was called, on the 25th of October, 1876, to see Mrs. W., aged twenty-nine, at a distance of fifteen miles from town. I arrived at 5 P.M., and found her completely anemic. There was not the color of blood in her face, lips or tongue. She says she is in the fifth month of pregnancy—has been flooding constantly for the last two and a half months, and occasionally sooner, and she says sometimes she loses as much in fifteen minutes as she would usually lose during a whole monthly period. Her appetite is good, bowels regular, no cough or other trouble, pulse 80. Upon external examination, I found the womb enlarged to about the usual size for a five month's pregnancy, but having an unusually round, smooth and uniform outline, with quite a soft, spongy feeling. There was no mammary enlargement nor change in color of areola. Vaginal examination showed the os dilated to the size of a quarter of a dollar, soft and spongy, and presenting internally an open space, above which was presented a rough, spongy mass, which seemed to be disconnected from the womb. And now the question arose: Was it a regular conception, or a polypoid tumor? The stethescope gave no evidence of fœtal circulation, but on the left side of the womb, in the lower border of the lumbar region, was a peculiar sound, resembling a strong respiratory murmur, and the maternal heart had no regular sounds, but a kind of blowing puff.

With these lights before me, I at once decided that nothing short of emptying the womb could save her, if, indeed, that could, and so informed her husband, and requested counsel in the case, when Dr. H. M. Williams was sent for. But as fifteen miles were to be traveled and back, I concluded to proceed in the case at once. I commenced the administration of tinct. ergot in drachm doses every hour and a half till four doses were given, by which time labor pains were fully established, and the hemorrhage stayed. At 4 A.M., the 26th, the womb was emptied of its contents, which came away all in one solid bulk, there being no placenta nor membranes of any kind left, no hemorrhage following, and the lady appearing in quite a hopeful condition. Dr. Williams arrived at 5 A.M., in time to see the mass which had been discharged. It consisted, externally, of a reddish, brown, liver-colored fibrus structure, with here and there a flake of fat as large as a silver half-dollar, and this mass was of unequal thickness, from one-fourth of an inch to one inch thick, and formed, when removed from the vagina, an uniform exterior, showing no particular maternal connection; and, upon opening it, the internal surface was smooth, containing the amniotic fluid, about one pint, or, perhaps, a little less, and a fœtus perfectly developed to time, but dead—the cord being very small, and *connecting directly with the inner surface of the sack, there being no placenta.*

